# Alternative Enzyme Inhibition Assay for Safety Evaluation of Food Preservatives

**DOI:** 10.3390/life13061243

**Published:** 2023-05-24

**Authors:** Elena N. Esimbekova, Anastasia A. Asanova, Valentina A. Kratasyuk

**Affiliations:** 1 Institute of Fundamental Biology and Biotechnology, Siberian Federal University, 660041 Krasnoyarsk, Russia; nastasia.asanova@gmail.com (A.A.A.); valkrat@mail.ru (V.A.K.); 2 Laboratory of Photobiology, Institute of Biophysics of Siberian Branch of Russian Academy of Science, 660036 Krasnoyarsk, Russia

**Keywords:** alternative bioassay, food safety, food additives, toxic effects, food preservatives, enzyme inhibition assay, sodium benzoate, potassium sorbate, sorbic acid

## Abstract

While food additives are widely used in the modern food industry and generally are important in maintaining the ability to provide food for the increasing world population, the progress occurring in this field is much ahead of the evaluation of their possible consequences for human health. The present study suggests a set of single- and multi-enzyme assay systems for revealing toxic effects of the most widely spread food preservatives, such as sorbic acid (E200), potassium sorbate (E202), and sodium benzoate (E211) at the primary molecular level of their interaction with enzymes. The assay is based on the inhibition of enzyme activity by toxic substances proportional to the amount of the toxicants in the sample. The single-enzyme assay system based on NAD(P)H:FMN oxidoreductase (Red) proved to be most sensitive to the impact of food additives, with the IC_50_ values being 29, 14, and 0.02 mg/L for sodium benzoate, potassium sorbate, and sorbic acid, respectively, which is considerably lower than their acceptable daily intake (ADI). No reliable change in the degree of inhibition of the enzyme assay systems by food preservatives was observed upon elongating the series of coupled redox reactions. However, the inhibition of activity of the multi-enzyme systems by 50% was found at a preservative concentration below the maximum permissible level for food. The inhibition effect of food preservatives on the activity of butyrylcholinesterase (BChE), lactate dehydrogenase (LDH), and alcohol dehydrogenase (ADH) was either absent or found in the presence of food preservatives at concentrations significantly exceeding their ADI. Among the preservatives under study, sodium benzoate is considered to be the safest in terms of the inhibiting effect on the enzyme activity. The results show that the negative effect of the food preservatives at the molecular level of organization of living things is highly pronounced, while at the organismal level it may not be obvious.

## 1. Introduction

Driven by the need for an improved price and shelf life, the food industry is developing food additives and finding new applications for existing ones. Nowadays, there are 363 different additives permitted in food in the European Union (EU), and those from E200 to E297 are preservatives. The most extensively used preservatives are potassium sorbate (E202), sorbic acid (E200), and sodium benzoate (E211). Food treatment with the preservatives leads to damage to some sites inside the bacteria, including the genetic material of the cell (DNA, RNA), as well as the cell membrane [1]. These preservatives can be found in commonly used food products such as soft drinks, fruit products, mayonnaise, pickled vegetables, cakes, cheese, etc. [2,3]. The US Food and Drug Administration (FDA) recommends that the content of benzoic acid in food should not exceed 0.1%, and for potassium sorbate the acceptable range is established to be from 0.1 to 0.3% [4]. In line with the approximations given by the Joint FAO/WHO Expert Committee on Food Additives, the acceptable daily intake (ADI) of sodium benzoate should not exceed 5 mg/kg of body weight, while the ADI for sorbic acid and potassium salt should not exceed 25 mg/kg of body weight [5]. Based on the rules in the Russian Federation, the maximum level of these substances in liquid products and drinks is 300 mg/L (Sanitary Rules and Regulations of the Russian Federation No. 2.3.2.1293-03, 2003).

Despite the fact that the standards for the use of food preservatives are fixed by law, the mechanisms of the influence of these substances on human health are intensively being studied [6]. Thus, multiple concerns have been expressed in the literature about the possible negative impact of benzoic and sorbic acids and their salts on the gastrointestinal tract. Benzoic acid and potassium sorbate were shown to affect the diversity and composition of the intestinal microbiota, also indirectly changing the permeability of the intestinal epithelium [7]. This is supported by another study where consumption of drinking water with a sodium benzoate concentration of 1% affected the gut microbial population and mucosal immunity of adult mice during the experimental period of 30 days [8]. A study by Dey [9] revealed oxidative stress, changes in the physiological parameters of the duodenum, significant degenerative lesions, and changes in the tissue architecture of the outer muscle layer of the duodenal wall in rats exposed to sodium benzoate at a level exceeding the FAO/WHO threshold by 2–8 times.

It was established that sodium benzoate, potassium sorbate, and sorbic acid can dissociate and be absorbed by diffusion through the small intestine into the portal vein, entering the liver cells [10]. Although the half-life of these preservatives shows that the human body can metabolize them, in some cases (e.g., liver and kidney abnormalities) they enter the body continuously in various modes without any metabolization [11]. Multiple cases of negative impacts of the studied food additives on the biochemistry and morphology of liver cells are described in the literature [12,13]. In 1977, the study by Gatley and Sherratt [14] showed that benzoates accumulate in the mitochondrial matrix of the liver and kidneys. Micromolar levels of benzoates are able to initiate the mitochondrial permeability transition in isolated liver mitochondria and uncoupling oxidative phosphorylation [6]. In addition, the analyzed nutritional supplements were found to easily cross the blood–brain barrier [6,15], and therefore, they can directly affect the functioning of neurons and synapses [15]. Children develop an allergy to sodium benzoate and it can also influence neurotransmission and cognitive functioning and can be associated with symptoms of hyperactivity, inattention, and impulsivity [15], and with a number of other negative consequences [2,3,15]. Potassium sorbate and sorbic acid consumption can lead to contact dermatitis [16].

This occurs because there are essential problems in the process of safety evaluation both of food additives and drugs and other consumer products that could potentially be ingested, such as toothpastes, lipsticks, etc. [2,17]. In fact, safety evaluation assays based on a variety of animal models used to study humans reveal disparate characteristics without capturing the effects on the human body [18,19,20].

Additionally, methods using animal research are considered to be unethical procedures and are not considered humane. Therefore, there is a growing demand worldwide for human-relevant approaches and testing methods. In 2019, the Environmental Protection Agency (EPA) proposed plans to eliminate all mammal testing by 2035 and the European Parliament approved a landmark resolution to phase out the use of animals for all scientific purposes in September 2021.

Alternative methods to animal models include biochemical or enzyme based tests, in vitro cell culture systems [21,22,23,24], and in silico computational biomodeling [25,26], with some of these being able to correctly identify 87% of agents which are harmful to human health [27].

The application of enzyme inhibition assays for estimating the potential substance toxicity seems quite justifiable, since every effect of a toxicant on a living organism begins at the molecular level. The assays are based on the ability of toxic substances to specifically inhibit the activity of various enzymes or enzyme systems, with the degree of inhibition of the enzyme activity by toxic substances being proportional to the amount of toxicant in the sample [28]. Enzyme systems of different complexity have selective sensitivity to various classes of toxic substances, which allows them to be used to study the mechanisms of toxicant effects on the functioning of individual elements of metabolic pathways [29]. Thus, the enzyme inhibition assays are convenient and fast tools which allow one both to estimate the activity of potential toxicants at the molecular level and to avoid unethical procedures.

This study presents a comparative analysis of the widely used food preservatives potassium sorbate, sorbic acid and sodium benzoate, on enzymes upon the transfer from simple assay systems (single-enzyme reactions) to more complex multi-component molecular systems (coupled enzyme reactions). For the analysis, we selected a number of key enzymes involved in various metabolic pathways, including proteolysis, respiratory chain functioning, alcohol metabolism, nerve conduction, and lactic acid fermentation. The objects under study were the following single-enzyme systems: NAD(P)H:FMN-oxidoreductase (Red), alcohol dehydrogenase (ADH), lactate dehydrogenase (LDH), butyrylcholinesterase (BChE) and trypsin (Try), as well as coupled enzyme systems: a two-enzyme system NADH:FMN-oxidoreductase and luciferase (Red + Luc) and the three-enzyme systems ADH + Red + Luc and LDH + Red + Luc.

## 2. Materials and Methods

### 2.1. Reagents

The following reagents were applied: tetradecanal (Merck, Darmstadt, Germany), NADH (Gerbu Biotechnik, Heidelberg, Germany), NAD^+^ (AppliChem, Darmstadt, Germany), FMN (Serva, Heidelberg, Germany), 95% ethanol (Medkhimprom, Novosibirsk, Russia), S-butyrylthiocholine iodide (S-BCh-I) (Merck, Schaffhausen, Switzerland), Nα-Benzoyl-L-arginine ethyl ester (BAEE) (Sigma-Aldrich, St. Louis, MO, USA), HCl (SigmaTek, Khimki, Russia), 5.5′-Dithiobis(2-nitrobenzoic acid) (DTNB) (Sigma-Aldrich, Taufkirchen, Germany), L-lactate (Fluka, Tokyo, Japan), sodium phosphate buffer pH 7.4, potassium phosphate buffer pH 6.8–8.0, and Clark and Lubs buffer pH 7.6.

The experiments were carried out using the following lyophilized enzymes: Red from *Vibrio fischeri*, 0.15 U/mL (Institute of Biophysics, Siberian Branch of the Russian Academy of Sciences, Krasnoyarsk, Russia); a mixture of high-purity enzymes: 0.15 U of Red from *Vibrio fischeri* and 0.5 mg of recombinant Luc *Photobacterium leiognathi* (Institute of Biophysics, Siberian Branch of the Russian Academy of Sciences, Krasnoyarsk, Russian Federation); BChE from equine serum, 900 U/mg (Sigma-Aldrich, St. Louis, MO, USA); ADH from baker’s yeast, 300 U/mg (Sigma-Aldrich, St. Louis, MO, USA); LDH from rabbit muscle, 600 U/mg (Sigma-Aldrich, St. Louis, MO, USA); and trypsin from porcine pancreas, 1300 BAEE U/mg (Sigma-Aldrich, St. Louis, MO, USA).

Solutions of ADH, LDH, and BChE were prepared using a 0.05 M potassium phosphate buffer, pH 8. To prepare the solution of Red + Luc, 5 mL of a 0.05 M phosphate buffer pH 7.0 was inserted into the flask with the lyophilized enzymes.

As analytes, we used three food preservatives: sodium benzoate (Sigma-Aldrich, Amsterdam, The Netherlands), potassium sorbate (Supelco, St. Louis, MO, USA), and sorbic acid (Sigma, Beijing, China). Solutions of each preservative were prepared at a concentration that corresponded to their complete solubility, and the optical density of the solutions was less than 1.5, to provide correct analysis. The stock solutions of the preservatives were diluted until the relative activity of the enzymes became close to their activity in the presence of a control solution (distilled water).

### 2.2. The Effects of Food Preservatives on the Activities of Single-Enzyme Systems

The effects of the food preservatives on the activity of the enzymes in the single-enzyme reactions were estimated as a relative activity using the formula A = (A_t_/A_c_)∙100%, with A_t_ and A_c_ being the enzyme activities in the presence of the analyte and in the control solution, respectively.

The activity of the NADH-dependent dehydrogenases was determined by spectrophotometry using the Warburg optical test [30]. The method is based on the difference in the absorption spectra of the reduced (NADH) and oxidized (NAD^+^) forms of nicotinamide adenine dinucleotide at 340 nm. The activity of Red was assessed by utilizing the following reaction mixture: 600 µL of the 0.05 M potassium phosphate buffer pH 7.0, 8 mU of Red, 20 µL of the 0.5 mM FMN solution, 100 µL of the 0.4 mM NADH solution, and 50 µL of distilled water (control) or the analyte solution. The reaction mixture for the ADH-catalyzed reaction consisted of 1 U of ADH, 500 µL of the 0.05 M potassium phosphate buffer pH 7.0, 5 µL of 95% ethanol, and 100 µL of the 0.4 mM NAD^+^ solution. The solution utilized in the reaction catalyzed by LDH contained 500 µL of the 0.05 M potassium phosphate buffer pH 8.0, 8 U of LDH, 50 µL of the 2 mM L-lactate solution, and 100 µL of the 0.4 NAD^+^ solution.

Ellman’s assay was employed to measure the activity of BChE [31], with S-BCh-I used as a substrate. The optical density of the solutions was assessed at a wavelength of 412 nm. To measure the activity of BChE, 800 µL of the 0.05 M potassium phosphate buffer pH 8.0, 80 mU of BChE, 1.8 mL of the 0.4 mM DTNB solution, and 100 µL of the 120 mM S-BCh-I solution, and 50 µL of the solvent (control) or the analyte solution were sequentially added to the spectrophotometer cuvette.

The trypsin activity was determined by observing alterations in the solution’s absorbance at 253 nm caused by the hydrolysis rate of its specific substrate, BAEE. The reaction mixture consisted of 490 µL of the 0.1 M Clark and Lubs buffer pH 7.6, 11 U of trypsin, 40 µL of 1 mM hydrochloric acid, and 460 µL of the 0.5 mM BAEE solution.

A spectrophotometer UVICON 943 (Kontron Instruments, Milan, Italy) was used to estimate the activities of the single-enzyme reactions.

### 2.3. The Effects of Food Preservatives on the Activities of Multi-Enzyme Systems

The design of the multi-enzyme systems is based on the conjugation of enzymatic reactions, where the product of one reaction serves as a substrate for the next reaction. The multi-enzyme system activities were assessed by measuring the alteration in the luminescence intensity when exposed to food preservatives compared to distilled water (control solution).

The impact of the analytes under study on the multi-enzyme reactions was evaluated by measuring the remaining luminescence intensity, which was calculated using the formula I = (I_t_/I_c_)∙100%, where I_t_ and I_c_ denote the calculated mean luminescence intensity values in the presence of the food preservatives or control solutions, respectively.

The inhibitory effect of the food preservatives on the multi-enzyme system activity and bioluminescence intensity was assessed by determining the value of the parameter IC_50_, which represents the concentration of the active ingredient that decreases the enzyme system activity by 50%.

The luminescence intensity of the coupled enzyme system was analyzed using the following reaction mixture: 5 µL of the Red + Luc mixture containing 0.5 µg of Luc and 0.15 mU of Red; 350 µL of the 0.05 M potassium phosphate buffer pH 6.8; 50 µL of the 0.5 mM FMN solution; 100 µL of the 0.4 mM NADH solution; 50 µL of the 0.0025% tetradecanal solution; and 50 µL of the distilled water (control) or the food preservative solution.

To analyze the ADH + Red + Luc three-enzyme system’s activity, the following reagents were used: 5 µL of the Red + Luc mixture containing 0.5 µg of Luc and 0.15 mU of Red; 350 µL of the 0.05 M potassium phosphate buffer pH 7.0; 50 µL of the 0.002% tetradecanal solution; 5 µL of 0.5 mg/mL ADH; 50 µL of the 0.4 mM NAD^+^ solution; 5 µL of 95% ethanol; 10 µL of the 0.5 mM FMN solution; and 50 µL of the control or the food preservative solution.

To examine the LDH + Red + Luc three-enzyme system’s activity, the following reagents were utilized: 5 µL of the Red + Luc mixture containing 0.5 µg of Luc and 0.15 mU of Red; 350 µL of the 0.05 M potassium phosphate buffer pH 7.0; 50 µL of the 0.0025% tetradecanal solution; 50 µL of the 0.5 mM NAD^+^ solution; 5 µL of 0.5 mg/mL LDH; 50 µL of the 2 mM lactate solution; 10 µL of the 0.5 mM FMN solution; and 10–50 µL of the control or the food preservative solution.

Bioluminescence was measured with a Lumat LB 9507 bioluminometer (Berthold Technologies, Bad Wildbad, Germany).

The data are presented as the mean and standard deviation of the mean (M ± m) with a sample size of n = 5. A two-tailed Student’s *t*-test was employed to compare the statistical significance between the two sets. *p* < 0.05 was deemed statistically significant. The results were statistically processed using the Origin 7.0 (OriginLab Corporation, Northampton, MA, USA) and EXCEL software packages (Microsoft, Redmond, WA, USA).

## 3. Results

**Figure 1:**
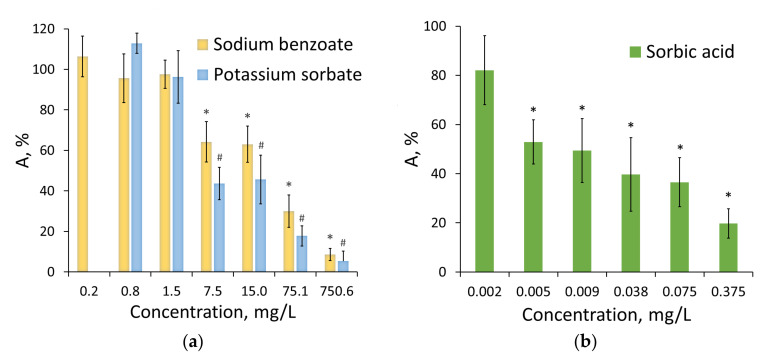
The effect of sodium benzoate and potassium sorbate (**a**) and sorbic acid (**b**) on the NAD(P)H:FMN-oxidoreductase activity. Data are presented as mean ± standard deviation of the mean (M ± m) with a sample size of n = 5. *^#^ *p* < 0.05 when comparing the relative activity values obtained in the presence of the distilled water used as a control solution.

The study considered the change in the activity of single- and multi-enzyme assay systems in the presence of food preservatives with respect to the control (distilled water). The food preservatives, namely potassium sorbate, sorbic acid, and sodium benzoate had different effects on the functioning of the single-enzyme systems. The strongest inhibiting effect demonstrated by the preservatives under study was exerted on the activity of Red (Figure 1); among the three examined preservatives, the enzyme was most sensitive to the effect of sorbic acid (Figure 1b). The IC_50_ values obtained for sodium benzoate and potassium sorbate were equal to 29 and 14 mg/L, respectively, which is three orders of magnitude higher than the IC_50_ value obtained for sorbic acid (0.02 mg/L).

**Figure 2:**
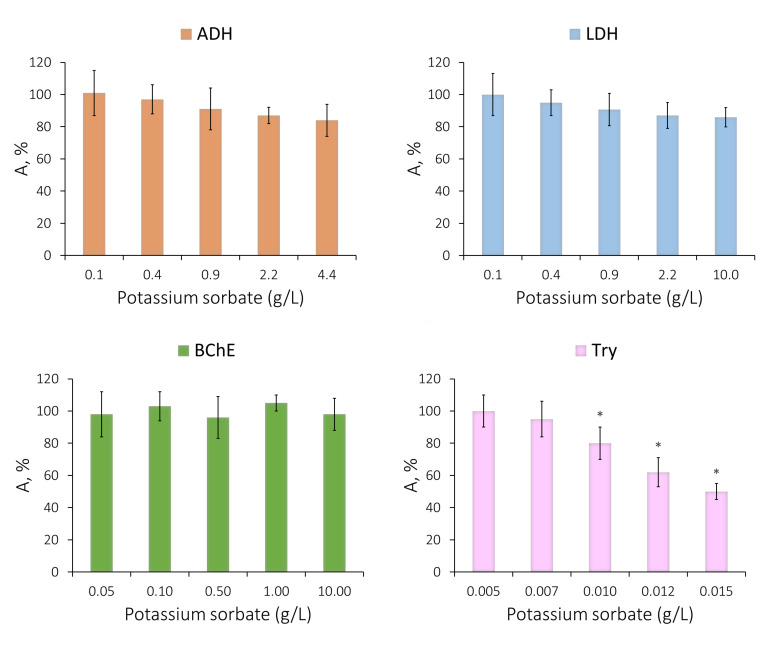
Relationship between residual activities of the single-enzyme systems and concentration of potassium sorbate. Data are presented as mean ± standard deviation of the mean (M ± m) with a sample size of n = 5. * *p* < 0.05 when comparing the relative activity values obtained in the presence of the distilled water used as a control solution.

There were no statistically significant differences in the change in the activity of ADH, LDH, and BChE, even in the presence of potassium sorbate at concentrations exceeding its ADI (Figure 2). Similar results were obtained in analyzing the effect of sodium benzoate on the enzyme activity. Statistically significant inhibiting effects were found for LDH in the presence of sodium benzoate (the IC_20_ value was 2.2 g/L) and ADH in the presence of potassium sorbate (the IC_20_ value was about 5 g/L). However, potassium sorbate had a considerable inhibiting effect on the Try activity, with the IC_50_ value being 15 mg/L, which is 20 times higher than its ADI in drinks (Figure 2).

As far as sorbic acid is concerned, due to its poor water solubility, its effect on the enzymes at concentrations not exceeding 50 mg/L was estimated. In the concentration range under study, sorbic acid had no inhibiting effect on the activity of ADH, LDH, or BChE.

**Figure 3:**
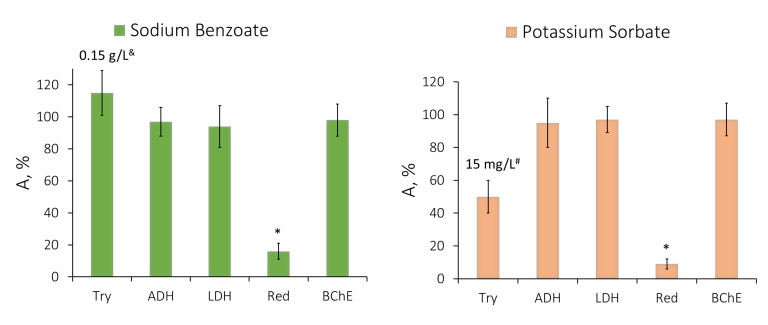
The effect of sodium benzoate and potassium sorbate at concentrations of 300 mg/L, corresponding to their acceptable daily intake, on the activity of the single-enzyme assay systems: trypsin (Try), alcohol dehydrogenase (ADH), lactate dehydrogenase (LDH), and butyrylcholinesterase (BChE). ^&#^ It was impossible to study the effect of sodium benzoate and potassium sorbate at a concentration of 300 mg/L on the trypsin activity due to the high optical density of the preservative solutions at the wavelength of measuring the trypsin activity (253 nm). Data are presented as mean ± standard deviation of the mean (M ± m) with a sample size of n = 5. * *p* < 0.05 when comparing the relative activity values obtained in the presence of the distilled water used as a control solution.

A comparison was made of the sensitivity of the single-enzyme assay systems to the effect of sodium benzoate and potassium sorbate at a concentration of 300 mg/L, corresponding to the maximum level of these substances in liquid products and drinks [5]. In the presence of the indicated preservative concentration, no change in the activity of ADH, LDH, or BChE was observed (Figure 3), with the Red enzyme activity decreasing by more than 80%. It was hardly possible to test the trypsin activity in the presence of potassium sorbate and sorbic acid at a concentration of 300 mg/L since both preservatives have high optical density in the wavelength range close to the wavelength used for the analysis of the trypsin enzyme activity (253 nm). Thus, the maximum concentration of potassium sorbate and sorbic acid for which the trypsin activity in their presence could be analyzed was not higher than 15 mg/L. A sodium benzoate concentration of 150 mg/L slightly stimulated the trypsin activity (Figure 3). Similar results were obtained in analyzing the effect of sorbic acid on the single-enzyme assay systems: this preservative had no effect on the activity of LDH and BChE, slightly inhibited the effect of ADH, and significantly decreased the activity of Red.

Further, the study involved testing how the enzyme sensitivity to the effect of food preservatives changed from simple single-enzyme reactions to complex multi-enzyme ones, including several coupled enzyme reactions, with the two-enzyme system Red + Luc and three-enzyme systems ADH + Red + Luc and LDH + Red + Luc.

**Figure 4:**
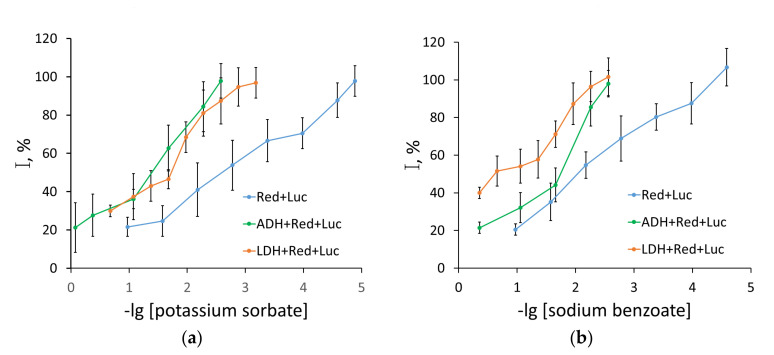

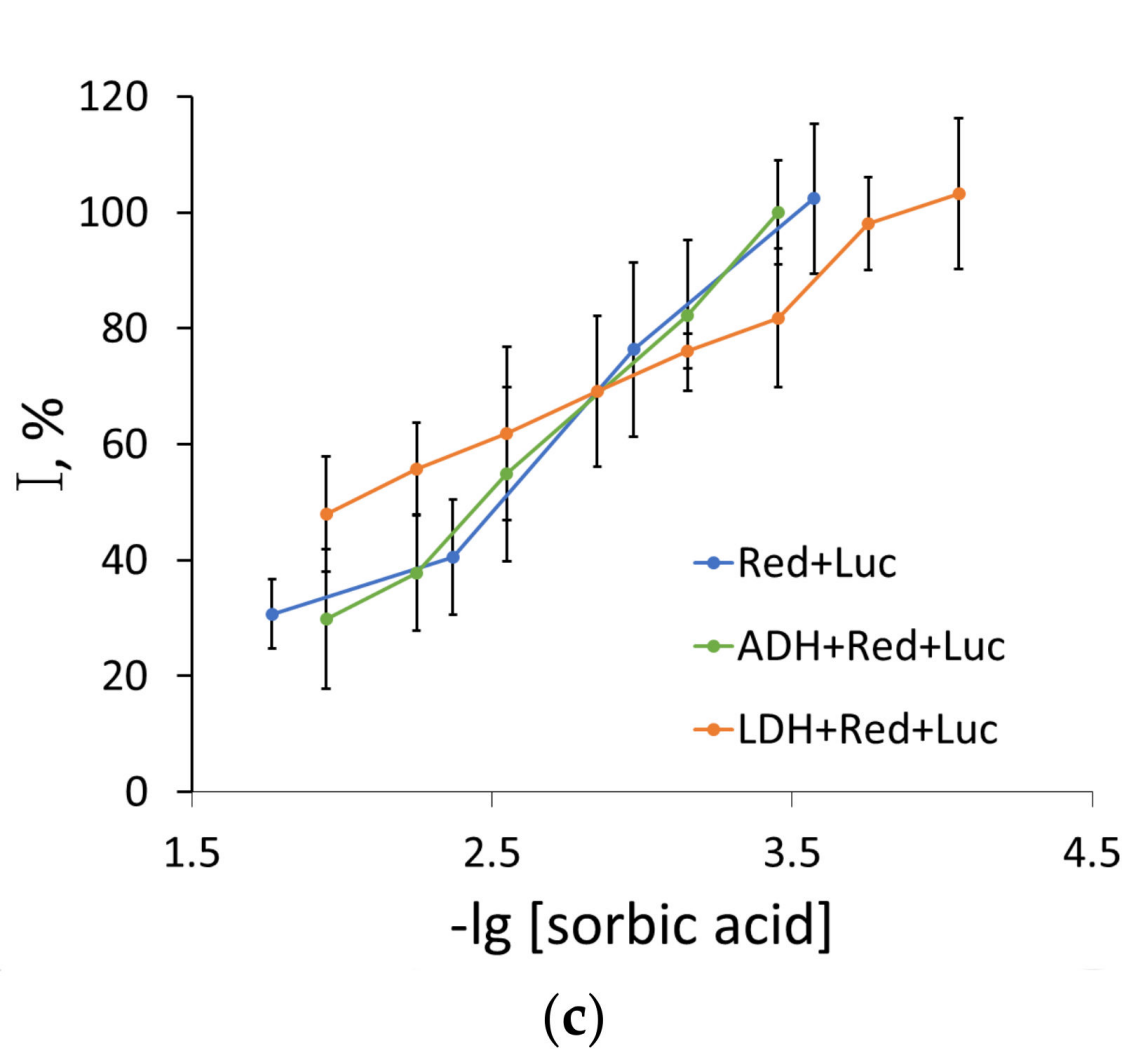
Relationship between residual luminescence intensity (I) of the two-enzyme system NADH:FMN-oxidoreductase and luciferase (Red + Luc), and three-enzyme systems with alcohol dehydrogenase (ADH + Red + Luc) and lactate dehydrogenase (LDH + Red + Luc) and the preservative concentration in inverse logarithm (−lg): (**a**) sodium benzoate, (**b**) potassium sorbate, (**c**) sorbic acid. The preservative concentration is given in g/L. Data are presented as mean ± standard deviation of the mean (M ± m) with a sample size of n = 5.

It was statistically proven from this study that the two-enzyme assay system Red + Luc (Figure 4) is the most sensitive to the effect of sodium benzoate and potassium sorbate. The sensitivity to the preservatives increased in the following sequence: sodium benzoate < potassium sorbate < sorbic acid, with the IC_50_ values being 10, 2.9, and 1.4 mg/L, respectively. The three-enzyme assay system LDH + Red + Luc also demonstrated high sensitivity to the effect of sorbic acid, with the highest IC_50_ value obtained for sodium benzoate, 255 mg/L, which is, nevertheless, lower than its ADI in drinks. Sorbic acid also had the highest inhibiting effect on the three-enzyme assay system ADH + Red + Luc (Figure 4), with the IC_50_ value being 2.9 mg/L, which is an order of magnitude lower than the corresponding values for sodium benzoate and potassium sorbate (Table 1). Therefore, for the multi-enzyme assay systems, the obtained IC_50_ values for all the three preservatives proved to be lower than their ADI.

**Figure 5:**
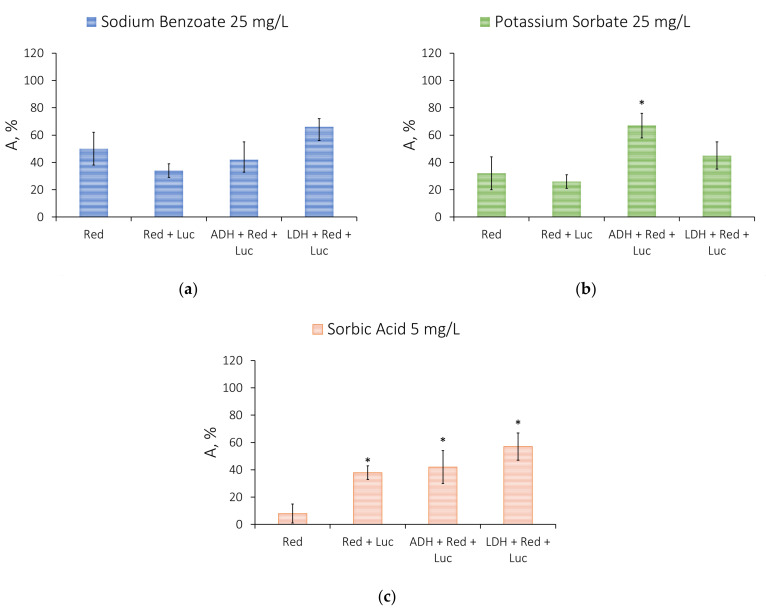
A comparison of the effects of the food preservatives on the activity of the enzyme systems with an increased length of the series of coupled reactions: (**a**) sodium benzoate at a concentration of 25 mg/L, (**b**) potassium sorbate at a concentration of 25 mg/L, (**c**) sorbic acid at a concentration of 5 mg/L. Data are presented as mean ± standard deviation of the mean (M ± m) with a sample size of n = 5. * *p* < 0.05 when comparing the relative activity values obtained for the Red assay system in the presence of the preservative solution. Red—NAD(P)H:FMN-oxidoreductase, Red + Luc—NADH:FMN-oxidoreductase and luciferase, ADH + Red + Luc—alcohol dehydrogenase, NAD(P)H:FMN-oxidoreductase and luciferase, LDH + Red + Luc—lactate dehydrogenase, NAD(P)H:FMN-oxidoreductase and luciferase.

Figure 5 shows the following statistically confirmed changes in the sensitivity of the assay systems upon increasing the length of the series of coupled reactions: Red → Red + Luc → ADH + Red + Luc and LDH + Red + Luc in the presence of the specified concentration of the food additives under study. The increased length of the series of coupled enzyme reactions is not found to result in an enhanced inhibiting effect of the preservatives. On the contrary, the three-enzyme assay systems are mainly less sensitive to the effect of preservatives as compared to the single-enzyme assay system based on Red. A highly significant difference was observed in the action of sorbic acid on the enzyme systems of various complexity. For example, in the presence of 5 mg/L of sorbic acid, the relative activity of the single-enzyme assay system with Red was about 5%, while the activity of the multi-enzyme assay systems in the presence of the given sorbic acid concentration was no lower than 40% (Figure 5c).

When comparing the IC_20_ and IC_50_ values (Table 1), one can conclude the existence of a significant difference in the effect of the preservatives under study on the enzyme assay systems, both from the absence of any effects (the single-enzyme system with BChE) or slight inhibition in the presence of extremely high preservative concentrations (single-enzyme assay systems with ADH and LDH) and from the inhibition of the enzyme activity even in the presence of the preservative concentrations which are significantly lower than their ADI (single-enzyme assay systems with Red or Try). The greatest inhibiting effect on the enzyme assay systems was exerted by sorbic acid. No inhibiting effect of the preservatives was observed upon increasing the length of the series of coupled enzyme reactions. Nevertheless, the obtained IC_20_ and IC_50_ values indicate the significant effect of the preservatives on the functioning of the enzyme systems of various complexity, even in cases where their concentrations are much lower than their maximum permissible levels for food and drinks.

## 4. Discussion

To date, consumers have raised concerns about the use of preservatives in food [3,15,32], since negative consequences have been reported associated with excessive consumption of food additives. Here, a comprehensive study was conducted to analyze the effect of food preservatives, namely potassium sorbate, sorbic acid, and sodium benzoate, on the functioning of enzyme assay systems of varying complexity. Of all the single-enzyme systems, the Red and Try assay systems were the most sensitive to the effect of food preservatives. Thus, we proved the inhibitory effect of potassium sorbate on the activity of the enzyme trypsin, secreted into the duodenum, at concentrations significantly lower than its established limit for food. This fact is consistent with previously obtained data, in which the trypsin activity was determined using the bioluminescent method by the rate of decrease in the luminescence intensity of the Red + Luc two-enzyme system of luminous bacteria [33]. Due to the limitations imposed by the trypsin activity determination method used in this study, it was not possible to determine the effect of sorbic acid on the functioning of the single-enzyme assay system with trypsin (the concentrations used did not exceed 5 mg/L). However, sorbic acid was previously shown to further inhibit the trypsin activity using the bioluminescent trypsin assay. The IC_50_ values for sorbic acid and potassium sorbate were calculated to be 1.6 and 12 mg/L, respectively [33]. A similar result was reported in [34], where the authors suggested that inhibition of the trypsin activity by potassium sorbate could occur due to the binding of the preservative to the enzyme in the region adjacent to the active center.

Regarding sodium benzoate, a very small effect of trypsin activation was found in the presence of a 150 mg/L solution of this preservative, which is also the maximum possible concentration that can be used for a correct analysis. The study by Mu and Liu [35] showed that the effect of sodium benzoate on the trypsin molecule is due to the disruption of the β-sheet structure of the protein and the release of internal amino acid residues, leading to a change in the tertiary and secondary structure of the protein molecule. This is confirmed by the results where the trypsin structure transformed from the β-sheet structure to the unordered coil structure upon interacting with butylparaben [36].

The trypsin activation by sodium benzoate is not a single example. Huo et al. [37] showed that sodium benzoate formed complexes with lysozyme, increased the polarity of the aromatic amino acid, effected the molecular skeleton of lysozyme, and stretched the secondary structure. Estimating the activity of lysozyme reveals that a 0.01 M solution of sodium benzoate increases the activity of the enzyme by more than 20%. However, there is evidence indicating that proteins can bind benzoate and then the benzoate-bound proteins enter the aggregation pathway to produce amorphous and fibrillar structures. It is important that the concentration of benzoates needed to induce protein aggregation is much lower than the microbicidal and inhibitory concentrations prescribed by the FAO [38].

In general, numerous studies have been devoted to the molecular effects of sodium benzoate. Moreover, this is associated with its application as a medicine rather than its application as a food additive. Indeed, sodium benzoate was shown to have neuromodulatory effects due to its ability to act as a competitive inhibitor of D-amino acid oxidase, one of the enzymes that regulate the levels of the endogenous ligand (D-serine) for the modulatory binding of glycine. Therefore, sodium benzoate is suggested for the treatment of patients with schizophrenia, as well as for the treatment of amnestic mild cognitive impairment and mild Alzheimer’s disease [15,39]. In mouse astrocytes, sodium benzoate was shown to abolish the suppression of the Parkin and DJ-1 proteins induced by the pro-inflammatory cytokine IL-1 beta, which counteract Parkinson’s disease [15,40]. However, here too everything is not so simple, for example, it was shown that sodium benzoate significantly impaired memory and motor coordination [41].

There is evidence in the literature that the consumption of sodium benzoate and potassium sorbate leads to complications in diabetes. For example, potassium sorbate enhances glycation and fibril formation of human serum albumin by non-covalent binding between the four amino acids Pro, Arg, His, and Arg [42]. In addition, it has been shown that potassium sorbate can have a side effect on human health through the activation of inflammatory pathways, causing an exacerbation of diabetes and triggering the gradual development of cancer [43]. However, there are conflicting data that indicate no adverse effects on insulin or glucose homeostasis [44].

In our work, it was sodium benzoate that had the least inhibitory effect on the single- and multi-enzyme assay systems. The only enzyme on which sodium benzoate had a significant inhibitory effect was Red. However, even Red is characterized by an increase in inhibitory action, in the sequence: sodium benzoate < potassium sorbate < sorbic acid. It should be noted that a decrease in the enzyme activity of Red by 50% occurs in the presence of food preservatives at concentrations much lower than its permissible content in food products.

It was shown that the inhibitory effect of food preservatives on the activity of BChE, LDH, and ADH was either absent or occurred at concentrations significantly exceeding their ADI. Thus, a decrease in the ADH activity by 20% was shown to occur in the presence of a 5 g/l solution of potassium sorbate, which is 16 times higher than its ADI. The proposed mechanism of the inhibitory effect of potassium sorbate on ADH lies in its ability to form covalent bonds between δ- and/or β-carbon atoms of the sorbate ion and the essential sulfhydryl group or ZnOH-group of the enzyme, as well as in the competition with ethanol and NAD^+^ for the binding site on the enzyme [45]. Similarly, sodium benzoate can only have an inhibitory effect on LDH if it is found to exceed the standards for its content in food products. BChE is an example of an enzyme which is not affected by any of the food preservatives under study.

Typically, enzyme reactions in a cell are organized into metabolic chains, where the product of one reaction is a trigger for starting the next enzyme reaction. Therefore, it is relevant to study the impact of toxic substances not only on the functioning of individual enzymes, but also on the series of coupled enzyme reactions, similarly to the processes occurring in the organism.

With an increase in the length of the series of coupled redox reactions in the multi-enzyme reactions Red + Luc, as well as ADH + Red + Luc and LDH + Red + Luc, there was no significant change in the degree of the effect of food preservatives on the functioning of enzymes as compared with the single-enzyme assay system based on Red. This is likely to be due to the lack of an inhibitory effect of the preservatives on Luc, ADH, and LDH. Thus, Red remains the only enzyme in the multi-enzyme assay systems whose activity is inhibited by the food preservative. Otherwise, we could observe an increase in the inhibitory effect of preservatives upon elongation of the series of coupled enzymes, as was the case when analyzing the inhibitory effect of a number of pesticides on enzyme assay systems of varying complexity [29]. However, an inhibition of activity of the tri-enzyme systems by 50% was observed at concentrations of the preservatives below the established maximum permissible content in food products. Moreover, the strongest inhibitory effect on the assay systems under study was again exerted by sorbic acid.

At present, quite a number of preclinical models for assessing the safety of potentially toxic compounds have been developed. For example, methods were proposed for growing cells on small devices to mimic organs or tissues, such as the intestine [21], liver [22], BBB [23], skin [24], and others. However, despite numerous positive results in tissue culture technologies, this area still faces many biological engineering problems associated with the search for biocompatible materials. In addition, these models include only a few cell types, and, therefore, cannot capture the full complexity of entire tissues or organs. Unlike in vivo assay systems, the set of single- and multi-enzyme assay systems proposed in this work can be considered as simple highly sensitive tools for detecting toxic effects of various substances at the primary molecular level of interaction, when the effects at the tissue or organism level can be not evident or mediated by other effects.

## 5. Conclusions

The preservatives sodium benzoate, potassium sorbate, and sorbic acid, which are intensively used in the food industry, have a significant inhibitory effect on a number of key enzymes that perform important functions in the human body. The greatest effect was shown to be exerted on the single-enzyme assay systems with Try or Red and multi-enzyme assay systems including Red. Sorbic acid was found to have the greatest inhibitory effect on the assay systems under study. The results show that the negative effect of the food preservatives at the molecular level of organization of living things is highly pronounced, while at the organismal level it may not be obvious. The proposed approach clearly demonstrates the prospects of creating a set of single- and multi-enzyme assay systems that allow the evaluation of the effect of existing and newly developed substances on the human organism for the upper threshold of their consumption to be identified.

## Figures and Tables

**Table 1:** The IC_20_ (mg/L) and IC_50_ (mg/L) values were determined from the effects of the food preservatives on the single- and multi-enzyme assay systems ^a^.

Enzyme Assay System	Sodium Benzoate		Potassium Sorbate		Sorbic Acid	
	IC_50_	IC_20_	IC_50_	IC_20_	IC_50_	IC_20_
Single-enzyme assay systems						
Red	29 ± 4 *	3.6 ± 0.6 *	14 ± 2 *	1.4 ± 1.9 *	0.020 ± 0.003 *	0.003 ± 0.0005 *
LDH	-	2200 ± 321	-	-	-	-
ADH	-	-	-	>5000		
Try	-^b^	-^b^	15 ± 2 *	10 ± 1.2 *	ND	ND
Multi-enzyme assay systems						
Red + Luc	10 ± 2 *	0.4 ± 0.1 *	2.9 ± 0.4 *	0.07 ± 0.01 *	1.4 ± 0.2 *	0.8 ± 0.1 *
ADH + Red + Luc	21.6 ± 3.1 *	5.8 ± 1.0 *	60.5 ± 7.1 *	5.8 ± 0.7 *	2.9 ± 0.3 *	0.6 ± 0.1 *
LDH + Red + Luc	255 ± 31 *	18.7 ± 2.6 *	18.7 ± 2.2 *	5.8 ± 0.6 *	10 ± 1.3 *	0.4 ± 0.1 *

^a^ No effect of sodium benzoate, potassium sorbate, and sorbic acid on the single-enzyme system BChE was observed. ^b^ Slight activation of trypsin (no higher than 10–15%) was found in the presence of the sodium benzoate solution at a concentration of 150 mg/L, it was impossible to estimate the effect of high sodium benzoate concentrations due to the high values of optical density of its solutions at 253 nm (wavelength for the analysis of the trypsin activity). ND—not detected. Sorbic acid at a concentration lower than 5 mg/L had no effect on the trypsin activity; the introduction of higher sorbic acid concentrations did not allow any reliable analysis to be made due to the high optical density of the solutions. * An inhibitory effect was observed at a concentration less than the acceptable daily intake (ADI), *p* < 0.05. The single-enzyme assay systems: Red—NAD(P)H:FMN-oxidoreductase, ADH—alcohol dehydrogenase, LDH—lactate dehydrogenase, Try—trypsin. The multi-enzyme assay systems: Red + Luc—NADH:FMN-oxidoreductase and luciferase, ADH + Red + Luc—alcohol dehydrogenase, NAD(P)H:FMN-oxidoreductase and luciferase, LDH + Red + Luc lactate dehydrogenase, NAD(P)H:FMN-oxidoreductase and luciferase.

## Data Availability

Not applicable.
